# Surgical approach for complete resection of giant retroperitoneal liposarcoma with diaphragmatic hernia via ninth rib thoracotomy

**DOI:** 10.3389/fonc.2023.1239962

**Published:** 2023-08-23

**Authors:** Bai-e Hu, Chang-lei Wu, Ji-peng Liu, Wen-jun Zhang

**Affiliations:** ^1^ Department of Rehabilitation Medicine, The Second Affiliated Hospital, Nanchang University, Nanchang, Jiangxi, China; ^2^ Department of Physical Examination, The Second Affiliated Hospital, Nanchang University, Nanchang, Jiangxi, China; ^3^ Department of Gastrointestinal Surgery, The Second Affiliated Hospital, Nanchang University, Nanchang, Jiangxi, China

**Keywords:** retroperitoneal liposarcoma, diaphragmatic hernia, approach, treatment, surgery

## Abstract

**Background:**

Resection of a giant retroperitoneal liposarcoma is difficult and technically demanding, especially for large retroperitoneal tumors accompanied by a diaphragmatic hernia. Technically, the open abdominal approach can be time-consuming and difficult to perform, with possible intraoperative complications and other factors bringing psychological and physical difficulties to the patient. This study reports a safe and feasible approach for the complete resection of a large retroperitoneal tumor complicated by a diaphragmatic hernia.

**Methods:**

A 58-year-old male patient with persistent upper abdominal pain and distension was treated at a local hospital on 4 July 2022. Computed tomography showed a mixed-density mass on the right retroperitoneum, and liposarcoma was considered. On 6 July 2022, the patient was transferred to our hospital for further treatment. Computed tomography showed a mass with low-density fatty shadow in the right adrenal region. The boundary with the right adrenal gland was unclear. The mass was 102 mm × 74 mm, and the right lobe of the liver was compressed. Insufficiency of the right middle lobe of the liver was seen due to a right diaphragmatic hernia and left mediastinal deviation. We considered the traditional approach for tumor resection via laparotomy, but we opted to perform a comprehensive evaluation first. The tumor was close to the back of the right kidney and liver, causing the diaphragm to rise because of its proximity to these organs. Exposing the tumor through laparotomy would be difficult, making it challenging to remove. The patient had a diaphragmatic hernia and moderate pulmonary dysfunction; therefore, we decided to enter the abdomen through a thoracotomy of the ninth rib.

**Results:**

Using our technique, the tumor was easily visualized and completely removed in approximately 30 min. The intraoperative blood loss was 100 ml, and no postoperative bleeding, pneumothorax, intestinal fistula, infection, or other complications occurred.

**Conclusion:**

The transthoracic approach may be a safer and more feasible resection method than the traditional open approach for patients with giant retroperitoneal liposarcoma with a diaphragmatic hernia.

## 1 Introduction

Retroperitoneal tumors occur in the peritoneal space and originate from tissues such as fat, muscle, lymph, blood vessels, and nerves (1). Retroperitoneal tumors are relatively rare, especially giant retroperitoneal liposarcomas. These tumors can show expansile growth in their early stage with no apparent symptoms. Most symptoms are caused by the increase in tumor volume and compression of surrounding tissues, requiring patients to seek medical attention (2). Large retroperitoneal tumors, generally larger than 10 cm, are rare, especially when complicated by a diaphragmatic hernia. These large tumors are difficult to resect, can cause many symptoms in patients, and can impact breathing and blood flow. A skilled and experienced surgeon is required with clear requirements for the anatomy of the abdominal tissue to operate on these large tumors. When the tumor is large, spatial visualization concerning the surrounding tissue is difficult during the operation, posing certain challenges to the surgeon (3, 4). The traditional operation approach used to treat retroperitoneal liposarcoma requires entering the abdominal cavity via laparotomy and exposing the tumor and surrounding tissue from different angles. For large tumors, the surgery is more invasive and has added risks for the patients. For example, the space is smaller, and tissue injury and increased bleeding may occur (5, 6). Therefore, it is important to find a safe and feasible way to reduce the operation time, expose the tumor tissue for complete resection, and alleviate the patient’s symptoms. The traditional method of laparotomy to remove retroperitoneal liposarcoma presents surgeons with certain challenges. These challenges include long procedure times, increased bleeding, and the possibility of incomplete resection. In our case, the patient had retroperitoneal liposarcoma with a diaphragmatic hernia. A thoracotomy was considered after a comprehensive evaluation of the patient. The tumor was completely exposed during the operation, the procedural time was short, and the patient recovered well postoperatively.

## 2 Methods and results

### 2.1 Description of the patient’s condition

A 58-year-old male patient with persistent pain in the upper abdomen and abdominal distension after eating presented to a local hospital on 4 July 2022. The patient had no nausea, vomiting, dizziness, fatigue, or other symptoms. Computed tomography (CT) showed a defect in the right diaphragm, herniation of the right inferior bowel, mesentery, gallbladder, and part of the liver into the right thoracic cavity. A mass of mixed density was observed in the right retroperitoneum, approximately 102 mm × 74 mm, with clear edges. An additional scan showed moderate enhancement in the parenchyma, compression of the right adrenal gland, and unclear visualization of the right kidney boundary. No obvious fluid in the abdominal cavity was observed, and liposarcoma was first considered. However, it was decided not to perform surgery at that time, and the patient was discharged after being treated conservatively for abdominal pain.

**Figure 1:**
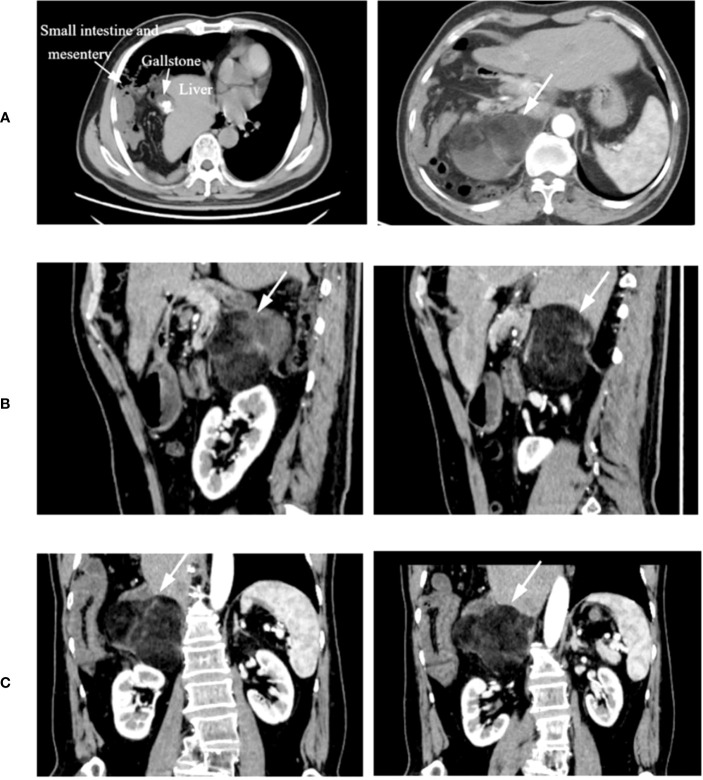
Representative CT images. **(A)** Cross-sectional CT showing part of the liver and small intestine and mesentery herniated into the thoracic cavity. **(B, C)** CT sagittal and coronal scans, with the tumor location indicated by the finger.

On 16 July 2022, a day after the patient was discharged from our hospital, CT showed a mass in the right suprarenal area near the right kidney, and it was highly likely to be a myeloid liposarcoma. Insufficiency of the right middle lobe of the liver was seen due to the right diaphragmatic hernia and left mediastinal deviation (**Figure 1**). Physical examination revealed the following: body temperature, 36.4°C; respiration rate, 20 breaths/min; blood pressure, 122/80 mmHg; pulse, 98 beats/min; and epigastric distention and tenderness, with no rebound tenderness. Other laboratory tests, including routine blood tests, biochemical tests (including liver and kidney function and electrolytes), and evaluation of the coagulation function were normal. Pulmonary function tests showed moderately restrictive ventilatory dysfunction, and maximum ventilation accounted for 51% of the expected moderate reduction. According to the CT, liposarcoma was the diagnosis, and we used that information to determine the best treatment options for the patient. At that time, we communicated with the patient and his family following the traditional surgical method protocol and planned to perform a laparotomy to remove the tumor.

However, we carefully evaluated the CT and the patient before surgery. Owing to the diaphragmatic hernia (caused by trauma in 1998), there was a diaphragmatic defect and some herniation of the abdominal organs into the right thoracic cavity. The tumor location was carefully evaluated, which was behind the right kidney and liver and close to the diaphragm. The visualization of the abdomen between the tumor and surrounding tissues was difficult. Resection could be difficult and time-consuming, with an increased risk of intraoperative bleeding and injury to the peripheral organs. Therefore, we opted to use the upper chest as the entry point to the abdomen through the diaphragm, which could better expose the tumor. Finally, the patient and his family were informed of the possible complications during and after the operation. After obtaining consent, the operation was performed using the ninth rib as the incision point, which reduced the procedural time and the impact on the patient.

### 2.2 Surgical intervention

**Figure 2:**
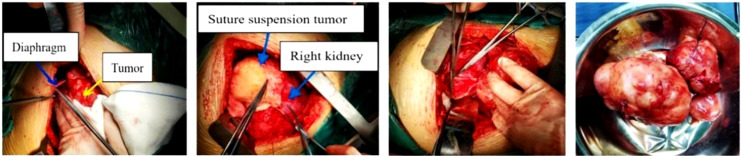
Intraoperative tumor resection.

Routine blood preparation was performed before surgery, and the patient was transferred to the operating room. The patient was in the left lateral decubitus position on the operating table, and the surgeon, nurse, and anesthesiologist checked the patient’s data. The patient received general anesthesia with endotracheal intubation. After successful anesthetizing, routine disinfection and draping were performed. An incision of approximately 20 cm in length was made at the ninth intercostal space. The skin, subcutaneous tissue, aponeurosis, muscle, and pleura were incised sequentially, and the bleeding point was carefully coagulated. Intraoperative exploration revealed that the liver herniated into the thoracic cavity, the right diaphragm was defective, and the bilateral diaphragms were atrophied. A mass of approximately 10 cm × 8 cm was seen above the right adrenal gland, which had adhered to the surrounding tissues. The adhesions around the mass were carefully separated, the bowel was opened to expose the mass, and the mass was abruptly dissected. Careful separation of the mass from the adhesions of the right kidney and adrenal gland did not damage the mass. The adhesions and nourishing blood vessels were gradually separated, and the blood vessels were sutured and checked for bleeding. The mass was slowly removed in its entirety, and a drainage tube was placed in the right abdomen (**Figure 2**).

**Figure 3:**
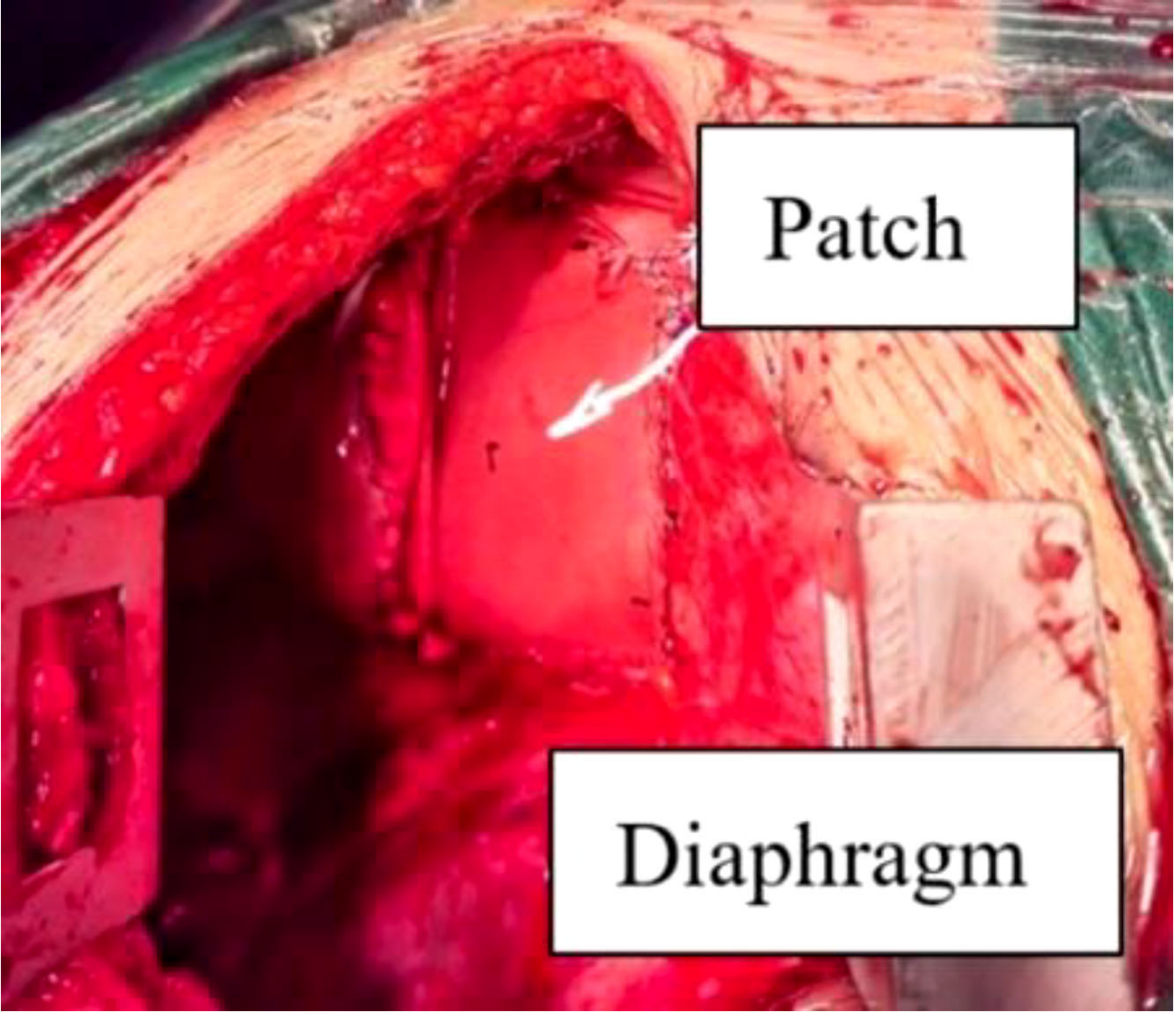
Diaphragmatic defect patch repair. After the tumor was completely removed, the patch was placed on the diaphragm defect and sutured to make up the defect and repair the integrity of the diaphragm.

The diaphragmatic hernia repair was the final part of the procedure. Visualization of the ninth rib in the thoracic cavity showed that the right lung and liver had adhered to the pleura, liver, and part of the intestine and had herniated into the thoracic cavity, making it difficult to repair the defect from the ninth rib incision. We decided to make a 20-cm incision in the seventh intercostal space to enter the chest and push the herniated thoracic organs into the abdominal cavity. We found the stumps on both sides of the diaphragm and sutured the edges of both ends with No. 7 silk thread for traction. A 15 cm × 9 cm patch (American Medtronic Self-Adhesive Patch, Pp1509g) was trimmed to fit the size of the diaphragm defect. The area around the stump of the diaphragm was sutured to repair the defect and restore the integrity of the diaphragm (**Figure 3**). After the patch was fixed and checked for active bleeding, the abdominal cavity was flushed with normal saline, and a chest drainage tube was inserted to connect to the water-sealed bottle. After checking the gauze, the chest cavity was closed layer by layer, and the lungs and water bottle were sealed again. After the fluctuations in the bottle were normalized, the operation was complete. The operation went smoothly; the procedure for tumor resection required approximately 30 min, and the intraoperative blood loss was 100 ml. Surgical specimens were sent for pathological examination after being inspected by family members. The patient was sent to the recovery room accompanied by the medical staff. Pathological examination revealed that the tumor comprised mature fat and bone marrow components, hemorrhage in some areas, and fibrous necrosis with hyalinization. The pathological diagnosis was myeloliposarcoma, with no tumor involvement at the resection margins.

### 2.3 Postoperative outcomes

**Figure 4:**
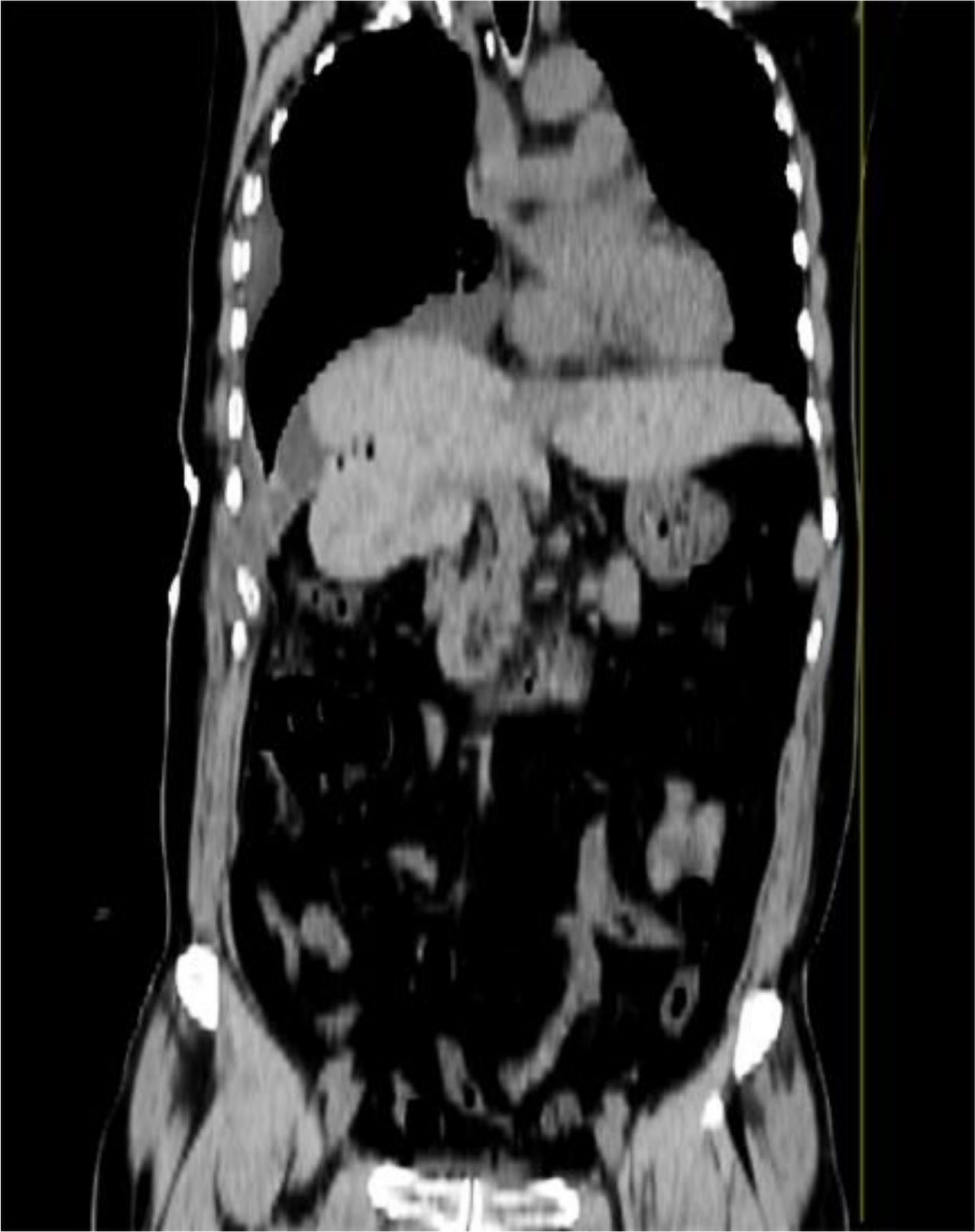
Representative images of abdominal CT after 10 days. Coronal CT showed that there was a small amount of fluid in the pleural cavity and that the liver had been repositioned into the abdominal cavity.

After the operation, the patient fasted and was administered fluid replacement, nutritional support, nebulization, anticoagulation, pain relief drugs, and other comprehensive treatments. Routine blood tests, liver and kidney function, and electrolytes were regularly checked. The patient recovered well after the operation; the chest and abdomen drainage tubes were unobstructed and gradually decreased, and there were no complications such as anastomotic leakage, abdominal distension, or pain. On postoperative day 10, the patient underwent CT to visualize the reduction of the abdominal organs and the changes in pleural effusion. The CT showed that the abdominal organs that herniated into the thoracic cavity had been reset, but a small amount of fluid in the thoracic cavity persisted (**Figure 4**). Because of the preoperative complexity of this case, pleural effusion always exists after tumor resection and repair of a diaphragmatic hernia. We recommended the patient be hospitalized to facilitate postoperative recovery and avoid complications. During postoperative management, the pleural effusion was gradually absorbed, and the thoracic drainage tube was removed. No other related complications were observed postoperatively. The patient recovered well, and the incision healed. The patient returned to a normal diet and was discharged on postoperative day 28.

## 3 Discussion

Liposarcoma originates from mesenchymal cells, occurs in adult malignant soft tissue sarcomas, and has multiple satellite lesions beyond its boundaries (7). Liposarcomas are usually divided into four subtypes: highly differentiated/atypical, dedifferentiated, myxoid/round cell, and pleomorphic liposarcoma (8). Dedifferentiated liposarcoma is more common in late adulthood and has no sex predisposition. The retroperitoneum is the most common site of liposarcoma (> 80% of cases). Other areas include the limbs, spermatic cord, torso, head and neck, and subcutaneous tissues (9). A study reported a dedifferentiated liposarcoma with a 3 cm × 2 cm diameter on the medial side of the left thigh in a 24-year-old woman (10). Leiomyoma is a rare myoma of the extremities that originates from smooth muscle cells and is mainly divided into skin, blood vessels, and deep soft tissue leiomyoma. However, myomas are more common in the lower extremities than in the upper limbs (11, 12). Therefore, the possibility of this type of lesion should be considered in the differential diagnosis of solitary pain with slowly growing masses in the extremities.

Giant retroperitoneal liposarcoma causes severe complications, such as abdominal pain, distension, and compression of adjacent tissues and organs. For those with a diaphragmatic hernia induced by a large tumor, it can affect breathing and blood flow, resulting in serious consequences (13, 14). Although chemoradiotherapy can reduce tumor size and slow tumor growth, there are side effects, and survival and recurrence rates also increase accordingly. Therefore, surgical resection is still the first and only way to cure retroperitoneal tumors (2, 15). In particular, for fast-growing malignant tumors, resection improves the survival rate and overall quality of life of patients. Postoperative radiotherapy and chemotherapy are options that can significantly improve the symptoms of patients if needed.

Giant retroperitoneal liposarcoma is a passive condition. If a patient has symptoms such as low protein and anemia, it is necessary to adjust their general health status to improve their suitability for surgery. However, in this case, the patient was well-nourished, without anemia, and could tolerate surgery well. Because of the relatively abundant blood supply of retroperitoneal giant lipoma, 1,000 ml–3,000 ml of blood should be prepared for the operation. Preoperative preparations for unexpected events during the operation are also necessary (16, 17). Resection of a retroperitoneal giant lipoma requires an experienced and skilled surgeon with a superior anatomical understanding of tissue structure. Choosing the appropriate incision site to expose the tumor tissue is crucial to improving the tumor resection rate. Because a large retroperitoneal liposarcoma occupies a large amount of space in the abdominal cavity, the surrounding tissues and organs are compressed, and the exposed space is small, making the operation difficult (18). Based on previous experience in retroperitoneal tumor resection, we found that the scope and selection of the surgical incision site are crucial to the complete resection of the tumor, which can reduce the operation time and the possibility of injury. While performing a comprehensive evaluation of the patient and the tumor, we abandoned the idea of the traditional transabdominal incision approach. We opted to use a method that could better expose the tumor. We entered the abdomen through the chest, and the tumor was visualized entirely and then removed. The postoperative results showed complete resection of the tumor, the operation time was short, and the intraoperative blood loss was small, thereby reducing the surgical impact on the patient and achieving satisfactory results. Exposing the tumor tissue through an abdominal incision is relatively difficult, usually taking 2–4 h or longer. In this case, complete tumor resection took only 30 min via the thoracic approach.

For the resection of large retroperitoneal tumors, attention should be paid to several aspects during surgery. First, the relationship between the tumor tissue and surrounding tissues and organs should be carefully evaluated, especially the large blood vessels, to determine whether it can be completely removed and to avoid damage and massive bleeding. In a large blood vessel injury, a gauze block can compress the bleeding. If the bleeding is considerable, fluid and blood can be transfused through the open channel simultaneously, and changes in vital signs, such as urine volume and blood pressure, can be closely monitored. Second, when peeling the tumor tissue, ensuring the integrity of its envelope, avoiding residual tumor tissue, and trying to achieve abrupt separation, working from the easy to the difficult separation without forcing separation, is of utmost importance. Attention should be paid to the existence of variable structures to avoid unnecessary damage during the operation. Large tumors that invade large blood vessels or surrounding tissues should not be rashly removed. Therefore, a better field of vision should be sought. Determining the spatial relationship between the tumor and surrounding tissues is particularly important. Palliative surgery or combined organ resection is possible. Finally, complete removal of the tumor tissue, checking for any remaining tumors, and complete hemostasis of the wound should be achieved. The abdominal cavity should be washed and soaked with chemotherapy drugs, washed with a large amount of normal saline, counted with gauze, and check again to confirm no bleeding. Importantly, although knot tying is a basic surgical technique, the need for firm and stable knot tying plays a significant role in preventing postoperative complications. In diaphragm repair and thoracic closure, if there are difficulties, it is recommended that a thoracic surgeon assist in suturing the diaphragm during closure to avoid the recurrence of diaphragmatic hernias and affecting the function of the diaphragm. Compared with a single abdominal incision or a right median incision, there are some disadvantages to making two 20-cm incisions through the chest. For example, it may increase the risk of pleural effusion, lung injury, and pneumothorax. It can also increase the incidence of postoperative pain, pulmonary infections, and incision-related infections and prolong the hospitalization and recovery time of patients. However, we comprehensively considered these factors during the preoperative evaluation. Given the overall condition of the patient and the prospect of better tumor exposure, complete resection, and diaphragmatic hernia repair, we implemented this feasible and safe method. Intraoperatively, the patient was given indwelling thoracic drainage. Postoperatively, the patient was given comprehensive care such as fluid replacement, nutritional support, anti-infection medications, atomization, tracheal management, and instructions to try small movements and resume their activities of daily living. It is suggested that a rehabilitation doctor be consulted and that lung and diaphragm function training be provided to patients. All of these aspects can optimize healing.

## Data availability statement

The raw data supporting the conclusions of this article will be made available by the authors, without undue reservation.

## Ethics statement

Ethical approval has been exempted by the Ethics Committee of the Second Affiliate Hospital of Nanchang University. The studies were conducted in accordance with the local legislation and institutional requirements. The participants provided their written informed consent to participate in this study. Written informed consent was obtained from the individual(s) for the publication of any potentially identifiable images or data included in this article.

## Author contributions

B-EH: Completed patient data collection and references. C-LW: Completed this data collation and drafted this article. J-PL: Completed the drawing and revised the references. W-JZ: Designed this study, completed the writing and revision of the article, and received funding. All authors contributed to the article and approved the submitted version.
